# Retracted: Myogenic regulatory factors are key players in determining muscle mass and meat quality in Jeju native and Berkshire pigs

**DOI:** 10.1002/vms3.793

**Published:** 2022-03-30

Kim, K, Kim, D, Min, Y, Jeong, D, Son, Y‐O, Do, K. Myogenic regulatory factors are key players in determining muscle mass and meat quality in Jeju native and Berkshire pigs. Vet Med Sci. 2021; 7: 735–745. https://doi.org/10.1002/vms3.418 The above article, published online on 29 December 2020 in Wiley Online Library (wileyonlinelibrary.com), has been retracted by agreement between the authors, the journal's Editor in Chief, Gayle Hallowell, and John Wiley & Sons Ltd. The retraction has been agreed at the request of the authors as a similar version of the same article has been published in the *Electronic Journal of Biotechnology* (1). The first author of this article and the corresponding author of the *Electronic Journal of Biotechnology* article were both working on a version of this research with the same Principal Investigator who has since passed away. Upon discussion between the two author groups, it has been agreed that the *Electronic Journal of Biotechnology* article is the original publication and therefore the decision has been made to retract this article.

(1) Simrinder Singh Sodhi, Neelesh Sharma, Mrinmoy Ghosh, Ram Saran Sethi, Dong Kee Jeong, Sung Jin Lee, Differential expression patterns of myogenic regulatory factors in the postnatal longissimus dorsi muscle of Jeju Native Pig and Berkshire breeds along with their co‐expression with Pax7, Electronic Journal of Biotechnology, Volume 51, 2021, Pages 8–16, ISSN 0717–3458, “https://doi.org/10.1016/j.ejbt.2021.03.001”

